# 

**Published:** 1912-10-26

**Authors:** 


### BUREAU OF INFORMATION.

### Rules for Correspondents.

1. Every letter, on whatever subject, must be accompanied by the coupon to be cut from the back cover (inside page) of The Hospital, current issue, and must contain the name and address of the correspondent with pseudonym for publication if desired.

2. Replies by post cannot be given, save under exceptional circumstances at the Editor's discretion.

3. Letters from Approved Homes in reply to special needs published in the Bureau should state terms and full particulars, and be sent prepaid under cover to the Editor of the Bureau with coupon and name enclosed for identification.

4. Proprietors of Homes which have not yet been entered on the List of Approved Homes, but have spare accommodation likely to suit special needs, are invited to write for an application form for registration. The fee for registration, which includes two announcements of the Home in the Bureau, is 5s.

5. Special needs of every description relating to those who are sick or in difficulties are made known in these columns free of charge.

6. All communications to be addressed to The Editor of The Hospital, 28 Southampton Street, Strand, London, W.C., and marked "Bureau of Information."

### Approved Homes.

Note.—*No Home is added to the List without direct medical and other testimony to its good management.*

**Isle of Wight.**—Landholm, Totland Bay. Proprietress: Miss Richardson. Medical, surgical, maternity, rest-cure, and other non-infectious cases. Massage as required. Very comfortable winter home at moderate terms, which can be specially reduced for ailing nurses.

**Stirlingshire.**—The Manse, Slamannan, N.B. Proprietress: Mrs. Reid. Slight mental or nerve cases. Incipient tuberculosis. Rural district, 500 feet above sea-level. Air very favourable for lung cases. Goat's milk if required.

### Homes Wanted.

**For Lady with Drug Habit.**—The patient is a lady who suffers from carbuncles, and is addicted to drugs. Kind care and supervision required. Payment not more than a guinea a week. Prepaid replies from Approved Homes forwarded to E. M. H. (126a).

**For Elderly Lady.**—The patient is eighty-six, and is at times feeble in mind as well as feeble in body, so that she requires care and attention. Terms 18s. to £1. Prepaid replies from Approved Homes forwarded to Mrs. B. (127a).

### Training and Employment.

**Medical Qualifications.**—It is quite impossible in this or in any other country to get qualified as a doctor by correspondence. Medical students must surrender their entire time for six or seven years. A few American so-called universities used to traffic in worthless "diplomas," but this has now been put a stop to, and at no time was the spurious qualification of any use to the deluded purchaser.—T. M., Rathgar.

**Desired Opening for Nursing Home.**—We have every sympathy with your wish to keep your children with you, but we cannot encourage you to hope for success under the circumstances. The profits on one patient would be far too small to enable you to run a house even with the aid of your small independent income, and we must warn you that the competition is exceedingly severe in this line, especially at the favourite seaside resorts within easy reach of London such as you suggest. The best hope would be to join with another nurse who already has the nucleus of a connection, and it is possible, if you decide on this plan, that we might be able to bring you into touch with some who would be glad to make you an offer.—Anxious.

**Plausible Agency.**—We are by no means favourably impressed with the circular you enclose. The guarantee "to effectually introduce patients or paying guests to your home or return your subscription in full" is merely a piece of bluff and, scattered broadcast to persons of whom the advertiser has no knowledge whatever, the promise has a singularly fallacious ring.—E. M.

### Nursing in the Empire and Abroad.

**Hospitals at Jerusalem.**—There are three British institutions in this town, viz. the London Jews' Society Mission Hospital, the British Ophthalmic Hospital, and St. Helena's Home, conducted under the auspices of the Anglican Bishop. Private nursing of a lucrative description does not exist, for visitors are mere birds of passage, and the place has every possible drawback for invalids. You might possibly succeed in obtaining a vacancy at one of the above institutions, but the staff in each case is very small.—Nurse Iota.

**Nursing Agencies in Venice.**—There are none. When British nurses are wanted, which is rarely, they are sent for from Florence. Italian nurses are paid about 4s. a day, and Italians cannot as a rule afford the higher terms required by British nurses.—Inquirer.

**Clapp Hospital, Zannit.**—We have received some charming photographs of this well-equipped hospital, for which hearty thanks. Please send us a copy of the annual report, with account of nursing staff.—Secretary.

**St. John's, Newfoundland.**—We are advised that the Colony is well supplied with nurses, chiefly American trained. Fees for private nurses vary from ten to fifteen dollars a week. It is quite probable if you joined your friends out there you would find a scope for your talents, especially as you have a midwifery certificate.—F. D. O.

### Letter-Box.

Beta.—Will let you have a full reply to your interesting queries next week.

Mrs. I.—We have forwarded your application for a district nurse to Sister Smith, 15 Buckingham Street, Strand, who will communicate with you direct.

Emm. Dee (124a).—Sorry none of the localities proved suitable; 900 feet above sea-level is not generally considered a high altitude. Will give you some addresses in France next week.

*** All who are sick or in difficulties have the opportunity of making their wants known free of charge under a pseudonym in the Bureau, and can thus be brought under the notice of those willing and able to render assistance. A large number of the Nursing Homes on the Approved List have generously signified their willingness to receive patients in poor circumstances recommended to them through the Bureau at greatly reduced terms. No charge is made on either side for the introduction of patients, and the advantages afforded by this free intercommunication between patients and homes of tried efficiency should be noted by all members of the medical profession.

**A leaflet containing particulars of the Bureau will be forwarded free on application.**

### Appointments.

Dr. H. R. Hutton, who has been on the visiting staff of the Manchester Children's Hospital for thirty years, having resigned, has been appointed consulting physician. Dr. Hugh T. Ashby, M.R.C.P.Lond., has been elected a visiting physician to the hospital. Mr. Edgar Grey, M.B., Ch.B., has been elected junior resident medical officer.

Mr. J. B. Howell, fourth assistant medical officer at Hanwell Mental Hospital, having resigned his appointment, Mr. S. Wyard and Mr. G. W. B. James, fifth and sixth medical officers, have been promoted to be fourth and fifth medical officer respectively, and Mr. J. F. O'Connell has been appointed sixth assistant medical officer.

The following appointments have been made at the Royal Free Hospital:—House physicians: Mr. B. Whitchurch Howell, M.R.C.S., L.R.C.P.; Miss H. H. Cuthbert, M.B., B.S. House surgeons: Mr. P. W. Ransom, M.R.C.S., L.R.C.P; Miss F. M. Edmonds, M.B., B.S. To be clinical assistants, gynæcological department: Miss Mary A. Blair, M.D., B.S.; and clinical assistant to Dr. Phear: Miss Middleton.

The following appointments have been made in the Hospitals of the Seamen's Hospital Society:—Mr. H. F. Holmden, M.B., Ch.B., Edin., and E. J. Storer, M.R.C.S., L.R.C.P., to be house physicians, and Mr. T. A. F. Tyrrell, M.B., B.S., Lond., and Mr. W. Archer Sneath, M.B., Ch.B., Vict., to be house surgeons at the "Dreadnought" Hospital, Greenwich; and Mr. J. T. Stewart, M.B., Ch.B., to be senior house surgeon, and Mr. W. F. Beattie, M.B., Ch.B., house surgeon, at the Albert Dock Hospital.

### KING EDWARD'S HOSPITAL FUND.

Among the latest contributions received for King Edward's Hospital Fund for London are the following annual subscriptions:—Army and Navy Co-operative Society (Limited), £105; Mr. Francis Reckitt, £52 10s.; Sir John Craggs, £25; Rev. A. W. Davies, £25; Messrs. Macmillan and Co. (Limited), £25; Goldsmiths' and Silversmiths' Company (Limited), £21; Mrs. Henry Hudson, £20.

